# Investigation of differences between chronological and vascular age in persons with multiple sclerosis

**DOI:** 10.1371/journal.pone.0311117

**Published:** 2024-11-20

**Authors:** Gorica D. Maric, Tatjana D. Pekmezovic, Olivera S. Tamas, Nikola D. Veselinovic, Maja S. Budimkic, Aleksa L. Jovanovic, Sarlota K. Mesaros, Jelena S. Drulovic

**Affiliations:** 1 Faculty of Medicine, Institute of Epidemiology, University of Belgrade, Belgrade, Serbia; 2 Faculty of Medicine, University of Belgrade, Belgrade, Serbia; 3 Clinic of Neurology, University Clinical Center of Serbia, Belgrade, Serbia; Heinrich-Heine-Universitat Dusseldorf, GERMANY

## Abstract

**Objective:**

To determine vascular age (VA) in a cohort of persons with multiple sclerosis (PwMS) in Belgrade, Serbia, and to assess the difference between chronological age (CA) and VA, in this population.

**Material and methods:**

A case-control study was conducted at the Clinic of Neurology, University Clinical Center of Serbia in Belgrade. Study participants (n = 274) were recruited during regular outpatient visits. Demographic and clinical characteristics including the presence of CVD comorbidities of PwMS were collected. Data were obtained using a questionnaire, designed and adapted for the study purposes. Additionally, fasting blood samples were collected from all participants, in order to determine their lipid profile. VA was calculated based on the patient’s sex, age, smoking status, total serum cholesterol level and systolic blood pressure (SBP) value. Afterwards, the study sample was divided into five groups with the different levels of the atherosclerotic burden, as follows: 1) PwMS without any CVD comorbidity; 2) PwMS with hyperlipidemia (HLP); 3) PwMS with HLP and hypertension (HTA); 4) PwMS with HLP, HTA and type 2 diabetes, and 5) PwMS with coronary artery disease (CAD). In the statistical analysis, for the determination of factors that are independently associated with the discrepancy between CA and VA in persons with MS, hierarchical regression analysis was performed.

**Results:**

The mean values of CA and VA were statistically significantly different among the groups(p<0.001). Additionally, a significant difference was also detected between CA and VA (p<0.001). The highest VA (66.4±15.8 years) and the difference between CA and VA (6.5±7.3 years) were registered only in the group comprising PwMS, HPL, HTA and type 2 diabetes. Results of the hierarchical linear regression analysis showed that the Expanded Disability Status Scale (EDSS) score, Body mass index (BMI), physical activity and the presence of type 2 diabetes, explained a total of 24% of the variations in the difference between CA and VA, in our cohort of MS patients.

**Conclusion:**

Our study showed significant difference between CA and VA in PwMS and additionally, increasing VA with atherosclerotic burden. Additionally, it has been demonstrated that crucial factors which led to the occurrence of these differences were BMI, physical activity, EDSS and the presence of type 2 diabetes.

## Introduction

Multiple sclerosis (MS) is the chronic inflammatory and neurodegenerative disease of the central nervous system (CNS) which affects 2.9 million people worldwide [1]. Although it has been generally accepted that first symptoms often present between the 20 and 40 years of age, growing evidence suggest an increasing incidence and prevalence in ageing persons with MS (PwMS) [2]. Currently, there are more than 20 approved disease modifying therapies (DMTs) for PwMS, but pivotal trials of the DMTs for relapsing MS included only patients younger than 55 years [3]. Therefore, the effectiveness of DMTs in older MS populations has not been fully clarified until now and warrants further research. Thus, it is important to point out that recently, multicenter randomized phase 2, non-inferiority trial including patients with MS of any subtype, 55 years or older, has been performed. The aim of this study was to determine the risk of disease recurrence in older patients who discontinue DMTs in comparison to those who remain on DMTs [3]. It has been concluded that stopping DMT might be acceptable in patients older than 55 years with stable MS, but a small increased risk of new MRI activity might exist.

Having in mind all above mentioned, comorbidity represents an important area in MS research nowadays, especially in older population, since it may have negative impact on different aspects of the disease, varying from the delayed diagnosis and treatment choice to disability progression and increased mortality [4]. Most common comorbidities in MS population are psychiatric and vascular disorders [5,6].

Similarly to the general population, cardiovascular diseases (CVDs) are among the leading causes of mortality in PwMS [7,8]. A systematic review with meta-analysis, including 160,000 PwMS, showed that standardized mortality ratio for CVD in MS population was 1.74 (95% Confidence Interval (CI) 1.67–1.81), as compared to the general population [9]. Also, a relationship between MS and vascular diseases was confirmed in several studies [10–13]. However, recently, it has been demonstrated that subclinical atherosclerosis was not associated with MS [14]. In this Canadian cross-sectional study, it was found that the proportion of study participants with subclinical atherosclerosis did not differ between patients with and without MS [14].

In order to quantify cardiovascular risk in an individual, different scoring systems were developed and majority of them were based on findings obtained in the Framingham Heart Study [15]. In 2008, a new scoring method was introduced, presenting the concept of vascular age (VA) [16]. It has been defined as the absolute cardiovascular risk according to the age of a person if all modifiable risk factors were controlled [16]. VA calculates the age of the patient’s blood vessels given its chronological age (CA), sex, total cholesterol (TC) level, smoking status and systolic blood pressure (SBP) [16]. Therefore, VA actually reflects atherosclerotic burden of the person. This means that persons of the same age can have different VA, depending on their cardiovascular risk, and also in one person CA and VA can be different. One of the main advantages of this method is that it may facilitate physician-patient communication [16].

Given the burden and impact of CVD comorbidity in PwMS, the aim of the present study was to determine VA in PwMS in Belgrade, Serbia, and to assess the difference between CA and VA, in this population.

## Material and methods

### Study design

A case-control study was conducted at the Clinic of Neurology, University Clinical Center of Serbia in Belgrade. All participants signed written informed consent prior to enrollment in the study. The study was approved by the Ethics Committee of the Faculty of Medicine, University of Belgrade (number—29/III-17).

### Selection of participants

MS patients, diagnosed according to the revised 2017 McDonald criteria [17], were recruited during regular outpatient visits, in the period November 4^th^, 2019 –May 29^th^, 2020, at the Clinic of Neurology, University Clinical center of Serbia, Belgrade. All participants were evaluated for the eligibility, for study inclusion. Inclusion criteria comprised fulfillment revised 2017 McDonald criteria [17], age 18 years and more, and willingness to participate in the study. Exclusion criteria were: age less than 18 years, heart failure and impaired renal and liver function, severe cognitive impairment and patients’ refusal to take part in the study. During the study period, a total of 274 MS patients met inclusion criteria. Based on the presence of the CVD comorbidity, study sample was divided into five groups of MS patients: 1) MS patients without any CVD comorbidity; 2) MS patients with hyperlipidemia; 3) MS patients with hyperlipidemia and hypertension; 4) MS patients with hyperlipidemia, hypertension and type 2 diabetes, and 5) MS patients with coronary artery disease. None of the patients in our sample suffered from type 1 diabetes.

### Measurements

Data were collected using a questionnaire, designed and adapted for the study purposes. The questionnaire included demographic (age and sex) and clinical characteristics (age at MS onset, age at MS diagnosis, usage of Disease Modifying Therapies (DMTs) and other treatment modalities). Data on the MS phenotype and the Expanded Disability Status Scale (EDSS) score [18] were extracted from patients’ medical records and from the population-based MS Registry, if needed. Additionally, in the questionnaire, variables regarding the presence of CVD comorbidities: hyperlipidemia, hypertension, type 2 diabetes, coronary artery disease, myocardial infarction, peripheral vascular disease, stroke and other CVDs, and CVD risk factors: smoking status, body mass index (BMI), family history of CVD, and physical activity, were included. Participants who reported having at least three moderate intensity, thirty minutes walks per week, were defined as physically active. Additionally, fasting blood samples were collected from all participants, in order to determine their lipid profile.

### Statistical analysis

In data analysis, descriptive and analytic statistics were used. For each participant, VA was determined according to the SCORE project tables for high-risk countries, given that Serbia is among the countries with the highest incidence and mortality from CVD [15]. VA was calculated based on the patient’s sex, age, smoking status, total serum cholesterol level and SBP value. Difference between CA and VA was calculated by subtracting CA from VA. Framingham risk score for estimation of 10-year CVD risk was determined using combination of patient’s sex, age, HDL-C, total cholesterol, systolic blood pressure, smoking status and presence of diabetes. Values <10% were considered as low risk, values ranging from 10 to 19% denoted intermediate risk and values above 20% indicated high risk of CVD during following 10 years [16].

Differences between groups for continuous variables were tested using the Tukey post hoc test, and for nominal variables via serial χ^2^ tests.

For the determination of factors that are independently associated with the discrepancy between CA and VA in persons with MS, hierarchical multiple regression analysis was performed. Predictor variables were separated into three blocks (models). Disability level, as measured by EDSS score, was entered in the first block; physical activity (yes/no) and BMI (second model) comprised the second block followed by type 2 diabetes in the third block. Data were analyzed using The Statistical Package for Social Sciences (SPSS), version 20.0. Probability level of <0.05 was considered statistically significant.

## Results

Table 1 presents demographic and clinical characteristics of the study participants stratified in groups according to the presence of comorbidities. The existence of the statistical significance between different groups is presented in the footnotes. Out of 274 patients, 59 of them were treated with ≥ 1 DMTs. Patients were treated with: interferon-beta preparations (N = 59), glatiramer-acetate (N = 11), teriflunomide (N = 4), dimethyl fumarate (N = 1), mitoxantrone (N = 6), S1PR modulators (fingolimod, N = 5; ozanimod, N = 1), natalizumab (N = 2), alemtuzumab (N = 1).

**Table 1:** Demographic and clinical characteristics of patients with multiple sclerosis.

Variable	Study group				
	MS without any comorbidity n = 106	MS and HLP n = 84	MS, HLP and HTA n = 41	MS, HLP, HTA and type 2 diabetes n = 27	MS and CAD n = 16
Proportion of females (%)	67.9	76.2	63.4	63.0	50.0
Chronological age (years)*^¶†§‡¥¤^	38.5±9.9	45.0±9.2	57.4±10.3	59.9±12.1	62.7±7.8
Vascular age (years)*^¶†§‡¥¤^	39.0±10.6	46.1±10.7	63.7±13.7	66.4±15.8	65.5±7.5
Difference between chronological and vascular age (years)^¶†‡¥£@^	0.5±1.0	1.0±2.2	6.3±6.6	6.5±7.3	2.8±3.7
Total cholesterol (mmol/L)*^¶†¥¤£^	4.3±0.6	6.1±1.0	5.8±1.3	5.1±1.1	5.0±1.2
Body mass index (kg/m^2^)^†¥^	22.9±4.1	23.5±4.1	25.1±4.0	27.4±2.3	25.8±5.5
Family history for CVD *N* (%)	86.7	88.1	96.0	92.3	100.0
Smoking *N* (%)	34.0	42.9	41.5	37.0	25.0
Physical activity *N* (%)^¶†§‡¥¤^	77.4	66.7	41.5	25.9	25.0
Age at MS diagnosis (years)^¶†§ ‡¥¤^	31.5 ± 11.1	34.3 ± 9.8	42.6 ± 11.3	48.2 ± 12.1	44.7 ± 11.6
Duration of disease (years) ^¶†§¤^	7.8 ± 6.8	11.1 ± 8.9	15.4 ± 11.5	13.6 ± 10.0	18.6 ± 11.4
MS phenotype - Relapsing remitting and secondary progressive - Primary progressive	65 (83.3%) 13 (16.7%)	55 (85.9%) 9 (14.1%)	31 (88.6%) 4 (11.4%)	16 (64.0%) 9 (36.0%)	11 (84.6%) 2 (15.4%)
EDSS (median, range) ^¶†¥¤^	3.0 (0–8)	2.5 (1–7)	4.0 (1–8)	6.5 (2–8)	5.5 (2.5–7.5)
DMT use • Yes • No	26 (25.2%) 77 (74.8%)	21 (26.3%) 59 (73.7%)	10 (25.6%) 29 (74.4%)	1 (3.8%) 25 (96.2%)	1 (6.7%) 14 (93.3%)
Statins treatment ^¶†‡¥^ • Yes • No	1 (0.9%) 105 (99.1%)	0 (0.0%) 84 (100.0%)	3 (12.0%) 22 (88.0%)	4 (30.8%) 9 (69.2%)	0 (0.0%) 2 (100.0%)
Antihypertensive treatment^¶†§‡¥¤β^ • Yes • No	3 (2.8%) 103 (97.2%)	7 (8.3%) 77 (91.7%)	23 (95.8%) 1 (4.2%)	9 (69.2%) 4 (30.8%)	2 (66.7%) 1 (33.3%)
Framingham risk score^¶†§‡¥¤^ • Low • Intermediate • High	67 (93.1%) 4 (5.6%) 1 (1.4%)	45 (84.9%) 6 (11.3%) 2 (3.8%)	5 (25.0%) 8 (40.0%) 7 (35.0%)	3 (37.5%) 1 (12.5%) 4 (50.0%)	0 (0.0%) 1 (100.0%) 0 (0.0%)

MS—multiple sclerosis; HLP–hyperlipidemia; CVD–cardiovascular diseases; HTA–hypertension; CAD–coronary artery disease; * statistically significant difference between MS without any comorbidity group and MS and HLP group; ¶ statistically significant difference between MS without any comorbidity group and MS, HLP and HTA group; † statistically significant difference between MS without any comorbidity group and MS, HLP, HTA and type 2 diabetes group; § statistically significant difference between MS without any comorbidity group and MS and CAD group; ‡ statistically significant difference between MS and HLP group, and MS, HLP and HTA group; ¥ statistically significant difference between MS and HLP group, and MS, HLP, HTA and type 2 diabetes group; ¤ statistically significant difference between MS and HLP group, and MS and CAD group; £ statistically significant difference between MS, HLP and HTA group, and MS and CAD group; @ statistically significant difference between MS, HLP, HTA and type 2 diabetes group, and MS and CAD group; β statistically significant difference between MS, HLP and HTA group, and MS, HLP, HTA and type 2 diabetes group.

**Fig 1:**
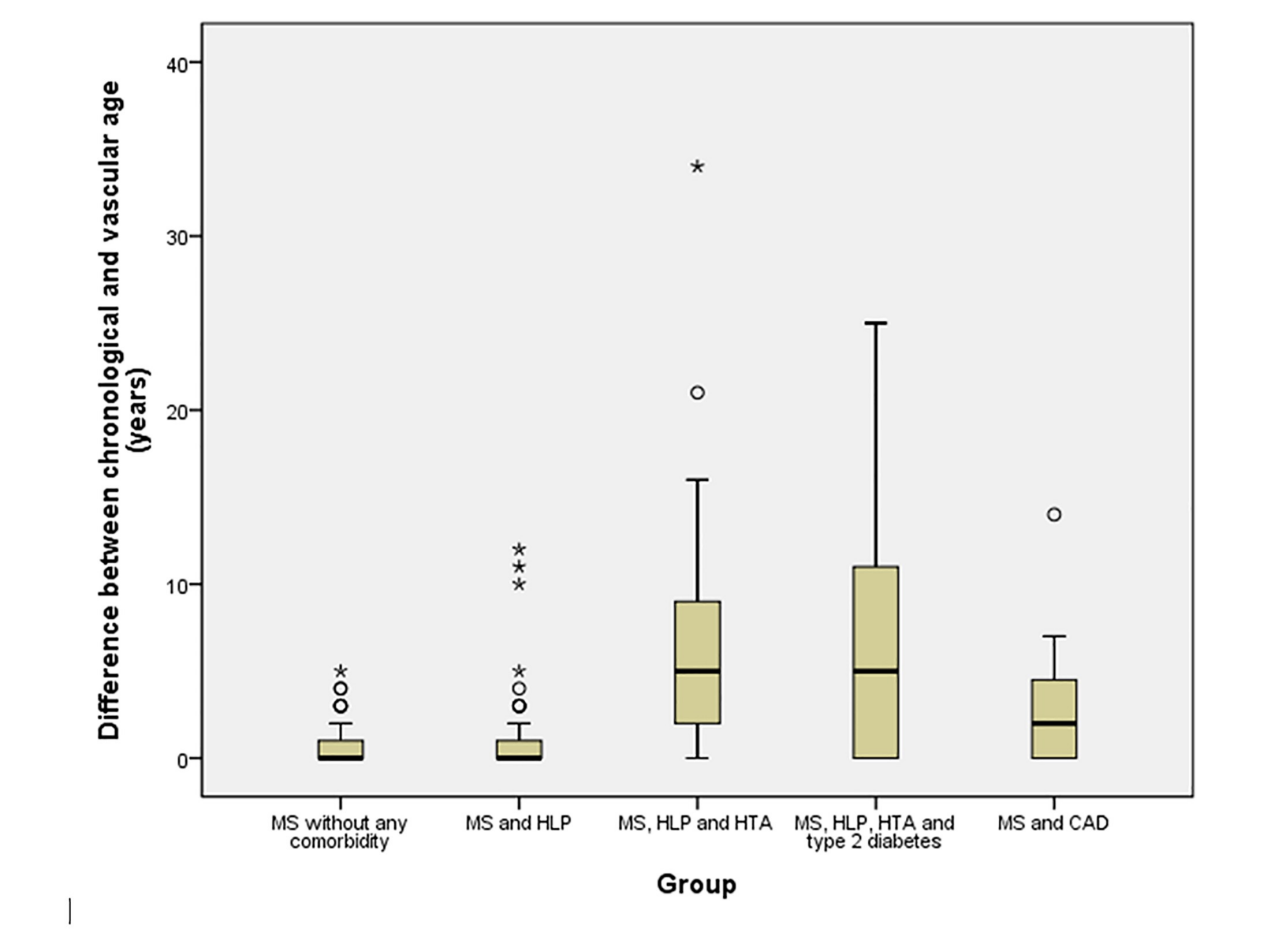
Difference between vascular and chronological age in the study groups. MS–multiple sclerosis; HLP–hyperlipidemia; HTA–hypertension; CAD–coronary artery disease.

The groups substantially differed among each other in the mean CA (p<0.001), VA (p<0.001) and in the difference between CA and VA (p<0.001). The highest VA (66.4±15.8 years) and difference between CA and VA (6.5±7.3 years) were registered in the group 4 (patients with MS, hyperlipidemia, hypertension and type 2 diabetes) (Table 1 and Fig 1). The values of VA had increasing tendency across the groups tested, with the rising number of CV risk factors, except, only the lower value detected in the group 5 (MS and coronary artery disease) compared to the group 4 (MS, hyperlipidemia, hypertension and type 2 diabetes). Data analysis revealed statistically highly significant differences in VA between group of MS patients without any CVD comorbidity and all other groups, as well as, between the group 2 (MS with hyperlipidemia) and all other groups. Groups 3, 4 and 5, didn’t differ significantly among each other, in terms of average VA values.

Table 2 shows results of the hierarchical linear regression analysis. In the first model, which included level of disability expressed as EDSS score, 7% of the variance of the difference between CA and VA was explained (*F* for change in *R*^*2*^ *=* 8.769, p<0.01). Second model explained additional 6% of the variance by adding the physical activity and BMI (*F* for change in *R*^*2*^ *=* 3.744, p<0.05). Finally, inclusion of the presence of type 2 diabetes in the third model, increased percentage of variance explained for another 11% (*F* for change in *R*^*2*^ *=* 16.246, p<0.01). Thus, EDSS score, BMI, physical activity and the presence of type 2 diabetes, explained a total of 24% of the variations in the difference between CA and VA in our sample of MS patients (Table 2).

**Table 2:** Summary of hierarchical regression analysis of variables predicting discrepancy between chronological and vascular age.

Variable	Model 1			Model 2			Model 3		
	Unstandardized B	Standard E (B)	Standardized β	B	SE (B)	β	B	SE (B)	β
EDSS	0.59	0.20	**0.26** **	0.56	0.25	**0.25** *	0.47	0.23	**0.21** *
Physical activity				-0.02	1.10	0.00	-0.46	1.05	-0.05
Body mass index				0.28	0.10	**0.24** **	0.19	0.10	0.16
Type 2 diabetes							-7.75	1.92	**-0.35** **
*R* ^*2*^	0.07			0.13			0.24		
*F* for change in *R*^*2*^	**8.769** **			**3.744** *			**16.246** **		

*p < 0.05

**p < 0.01.

## Discussion

The main findings of our study are that VA in MS population increases with atherosclerotic burden and that type 2 diabetes, EDSS score, BMI and physical activity, are responsible for 24% of the variance of the discrepancy between CA and VA in PwMS.

Both atherosclerosis, as underlying cause of vascular dysfunction, and MS have been shown to have inflammatory nature [14]. MS treatment could potentially play a significant role in modifying risk for vascular disease occurrence [19]. The impact of DMTs on cardiovascular comorbidities is extremely important, having in mind that the epidemiology of MS has shifted to an older than previously described population due to an improvement of outcomes with novel MS DMTs. Therefore, ageing with MS may lead to a longer exposure to DMTs and cumulative side effects of sequential DMTs. Additionally, older individuals are more prone to adverse effects of DMTs, especially those related to cardiovascular comorbidities [20]. For example, it is important to point out that sphingosine-1-phosphate (S1P) subtypes 1 to 3 are expressed on endothelial and vascular smooth muscle cells, contributing to the regulation of endothelial barrier function and peripheral vascular tone. Thus, fingolimod (sphingosine-1-phosphate receptor, S1PR), like endogenous S1P, may lead to a slight, transient reduction in blood pressure after initiation of fingolimod therapy in relapsing MS patients, followed by a small increase in both systolic and diastolic blood pressure. It has been described that this increase in blood pressure reaches a stable plateau after 6 months of treatment [21]. In clinical trials with another oral sphingosine-1-phosphate receptor 1 and 5 modulator, ozanimod, in patients with relapsing MS, both systolic and diastolic blood pressure slight increases, detectable 3 months after treatment initiation, have been demonstrated [22]. Teriflunomide treatment was also associated with increases in systolic and diastolic blood pressure in clinical trials in PwMS [23]. Therefore, further analysis of the potential impact of the exposure to DMTs, especially S1PR modulators and teriflunomide, on vascular age in patients with MS is warranted. We were not able to perform such an analysis, because the proportion of patients treated with these drugs in our MS cohort was extremely low. This was due to the fact that in our country, these drugs were available, being reimbursed, from May, 2020, after recruitment for this study had been finished.

Although we expected highest VA in the group of MS patients with greatest atherosclerotic burden (MS and coronary artery disease), this was not the case. The highest VA was observed in MS group with hyperlipidemia, hypertension and type 2 diabetes. However, this is maybe not surprising, having in mind that coronary artery disease, probably leads to the substantial changes in patients’ lifestyle. Patients with coronary artery disease and myocardial infarction are perhaps more prone to smoking cessation, regular physical activity, healthy diet and maintaining body weight within normal range, due to the severity of their medical condition. On the other hand, patients with coronary artery disease usually take lipid-lowering drugs, antihypertensive and other medications which can result in total cholesterol and systolic blood pressure values within normal range. Given that VA is calculated using total cholesterol level, smoking status and systolic blood pressure, it is possible to hypothesize that, due to above-mentioned reasons, mean VA in MS and coronary artery disease group was lower compared to the group 4 (MS, hyperlipidemia, hypertension and type 2 diabetes) and higher than that observed in other groups. Also, the largest discrepancy between CA and VA observed in the MS group with hyperlipidemia, hypertension and type 2 diabetes and not in MS and coronary artery disease group, could be similarly explained.

Hierarchical regression analysis results showed that the presence of the type 2 diabetes, physical activity, BMI and EDSS, are important factors that contribute to the magnitude of the gap between CA and VA. These findings are expected given their potential impact on atherosclerotic process. Previous studies have shown that PwMS could be more prone to subclinical atherosclerosis by measuring carotid intima-media thickness and revealing significant difference in CIMT between MS and non-MS population [19]. It is well-known that increased BMI, associated with low level of physical activity, leads to chronic inflammation state which in turn promotes atherosclerosis [19]. Additionally, with disability progression, PwMS become less physically active and have greater chance for the development of obesity, the association with atherosclerosis being very well established [7]. Although the presence of type 2 diabetes in PwMS is not as common as in general population, it has been suggested to have potential various negative influences on the MS course, resulting in the faster progression of disability and limited mobility [24–26].

Our study has certain limitations. First, it could have been beneficial if the total sample size had been larger. Furthermore, number of participants in each study group is not equal and is small in groups with the highest atherosclerotic burden. However, having in mind that the total Belgrade MS population comprises 2725 individuals, our sample represents almost 10%. Also, it should be stressed that some of the participants included in the study were treated with DMTs and it is well known that DMTs can alter inflammation status. Finally, we were not able to include certain biochemical parameters, such as HbA1c.

In conclusion, our study showed significant difference between CA and VA in PwMS and additionally, that VA increases with atherosclerotic burden. Furthermore, it has to be highlighted that important factors that drive these differences are BMI, physical activity, EDSS and presence of type 2 diabetes. Management of the comorbidity is one of the crucial steps in preserving brain reserve in PwMS [27]. It has been shown that early diagnosis of MS together with immediate treatment with DMTs, changes in the lifestyle and control of comorbidities, may significantly contribute to the brain health in this population and consequently to a slower disability progression and better MS outcome.
